# A prospective study of socio-demographic, clinical characteristics and treatment outcomes of children with tuberculosis in Sindh, Pakistan

**DOI:** 10.1186/s12879-019-3702-3

**Published:** 2019-01-24

**Authors:** Madeeha Laghari, Syed Azhar Syed Sulaiman, Amer Hayat Khan, Naheed Memon

**Affiliations:** 1 Department of Clinical Pharmacy, School of Pharmaceutical Sciences, Universiti Sains Malaysia, 11800 Minden Penang, Malaysia; 2 College of Pharmacy, Liaquat University of Medical and Health Sciences, Jamshoro, 76090 Pakistan

**Keywords:** Tuberculosis, Treatment outcomes, Pulmonary TB, Children ≤14 years, Pakistan

## Abstract

**Background:**

Understanding the explanations behind unsuccessful treatment outcomes in tuberculosis (TB) patients is important to improve treatment success. Treatment completion for TB is the mainstay of TB prevention and control. The study was aimed to assess the treatment outcomes and predictors for unsuccessful outcomes among children with TB.

**Methods:**

This was a prospective multicenter study conducted in Sindh. Children aged ≤14 years enrolled from June to November 2016 were included. A structured data collection tool was used to gather information with respect to patients’ socio-demographic, clinical and microbiological data. Additionally, to collect the information related to socio-economic and education level of caregivers, validated questionnaire was administered to the caregivers. Treatment outcomes were assessed according to the World Health Organization (WHO) guidelines. The relationship of unsuccessful treatment outcome with socio-demographic and clinical attributes of TB patients was analyzed using logistic regression model.

**Results:**

Childhood TB represented 19.3% (508/2634) of all TB cases in selected hospitals. Of these, 268/508 (52.8%) were females and one third of the children were aged ≤2 years (34.3%). In multivariate analysis, pulmonary smear positive TB (PTB+) (AOR = 5.910, 95%CI *=* 1.64–21.29), those with adverse drug reactions (AOR = 11.601, 95%CI *=* 4.06–33.12) and those who had known TB contacts (AOR = 3.128, 95%CI *=* 1.21–8.06) showed statistically significant association with unsuccessful treatment outcomes.

**Conclusions:**

The high proportion of childhood TB cases (19.3%) demonstrates the continuation of TB transmission in the study setting. Furthermore, an increased focus on PTB+ patients, those with adverse drug reactions and household contact with TB is warranted.

## Background

The principal objectives of TB treatment are to cure the individual with the disease and limit the transmission of *Mycobacterium tuberculosis* (*M. tuberculosis*) to others in the community [1]. The World Health Organization (WHO) suggests Directly Observed Therapy Short (DOTS) and has set curative thresholds of 85% [2]. The principle pointers used to measure advance in execution of DOTS are the availability of a National Tuberculosis Control Programme (NTP) manual consistent with the DOTS strategy, the number of administrative sectors in the country performing the new TB control system, the treatment cure and success rate in new smear positive cases, and the case detection rate [3].

In 2016, 51,000 (9.8%) of an estimated 518,000 TB cases notified in Pakistan, were children aged ≤ 14 years [4]. During the same year, a total of 41,758 cases of childhood TB were reported with the NTP [5]. There is evidence that in these settings most of the TB cases were being missed in large hospitals particularly in Tertiary Care Hospitals where most of the presumptive TB cases go unrecognized, the diagnosed and treated cases are not reported to the NTP or the treatment may not follow national guidelines [5].

In 2015, the treatment success rate in new and relapse cases in Pakistan was reported as 93% [4]. Treatment outcome at the end of therapy is an important marker of TB control programmes [6]. Moreover, examining and assessment of treatment outcomes of TB patients is an essential element of the DOTS strategy [7].

To the best of our knowledge, risk factors for poor treatment outcomes have not previously been evaluated among children in Pakistan. Furthermore, this was the first time that the rate and proportion of TB was studied among children at the study site. The study was conducted to highlight the issue of increasing trend of childhood TB. The objectives of the study were to describe the proportion of childhood TB cases, treatment outcomes and the possible risks of treatment failure in children aged ≤14 years.

## Methods

### Study settings and design

This prospective observational study of TB in children ≤14 years of age was conducted over a period of 18 months. The study was conducted in the following public sector hospitals:

i. Liaquat University of Medical and Health Sciences Civil Hospital, Hyderabad (LUMHS-CHH).
ii. Sindh Government Hospital Qasimabad, Hyderabad (SGH-QH).
iii. Shah Bhitai Government Hospital, Latifabad, Hyderabad (SBGH-LH).
iv. Jamshoro Institute of Chest Diseases Kotri, Sindh (ICDK).
v. Matiari Syed Baqadar Shah Taluka Hospital, Matiari (SBS-THM).

Children diagnosed with TB from 1 June 2016 through 30 November 2016 were consecutively enrolled and followed up monthly until the end of treatment. Children with drug-resistant TB and comorbidities were excluded. Verbal consent was sought from all guardians or caregivers (mostly parents) of patients.

### Data collection

A standardized data collection form was used to collect the information of patients regarding their socio-demographic, microbiological and clinical data. The socio-demographic variables included: age, gender, residence, body weight at baseline and monthly follow-up visits, level of education and monthly income of their treatment supporter and household contacts (HHC) with TB. Microbiological data consisted of sputum smear microscopy of acid fast bacilli (AFB) and Gene Xpert MTB/RIF (Xpert MTB/RIF) (Cepheid., New Jersey, USA) results at baseline. Clinical data contained category of TB at the start (new, relapse, lost to follow-up, failure), types of TB (pulmonary or extra-pulmonary), laboratory results and radiological findings of chest X-ray (CXR) at baseline and subsequent monthly visits (if recommended by clinician), Pakistan Pediatric Association (PPA) score, Bacillus Calmette-Guerin (BCG) scar, treatment regimen, adverse drug reactions (ADRs) and treatment outcomes of anti-tuberculosis Treatment (ATT). Body weight and age was used to determine weight for age using data table of weight for age charts of Centres for Disease Control and Prevention (CDC). Patients with weight < 5th percentile were recorded as underweight and those with ≥ 95th percentile weight for age were considered as overweight [8]. Logistic regression model was used to analyze the predictors for unsuccessful treatment outcomes. Factors found significant in univariate analysis, were included in multivariate logistic regression to estimate the odds ratios (ORs) with their 95% confidence intervals (CIs). *P* value < 0.05 was considered statistically significant. All the factors considered in the univariate analysis were based on literature review and suggestions from the clinical team.

### Diagnosis

Children were diagnosed as having TB considering suggestive clinical features, history of TB contact, a positive tuberculin skin test (TST) result, PPA scoring charts (suggested by Pakistan Paediatric Association) > 7 score and evidence of TB on CXR for pulmonary TB (PTB). Mycobacterial culture and Xpert MTB/RIF assays were used as add-on tests and specifically performed to exclude drug resistant-TB. Children presenting to the hospitals with clinical symptoms including cough lasting for ≥ 2 weeks, fever, night sweat, dyspnea, weight loss, sputum production and those indicative of extra-pulmonary TB (EPTB) were evaluated for TB. TST was done using standard of 5 Tuberculin Units (TU in 0.1 mL) (Sanofi Pasteur, Toronto, Canada), during the initial visit and read at 48–72 h. Since children were being evaluated for TB, a TST ≥ 10 mm induration was considered positive [9]. Fluorescence microscopy was used for sputum smear samples for the presence of AFB. In addition, Xpert MTB/RIF assay is routinely done in sputum smear negative and sputum smear positive patients for further confirmation of *M. tuberculosis* and drug-resistance TB based on NTP guidelines [10]. For EPTB, diagnostic tests were performed depending on the sites involved. CXR was done in all EPTB cases to exclude the pulmonary involvement. Fine needle aspiration cytology (FNAC) was the first-line diagnostic technique for the peripheral lymph nodes. However, if the FNAC examination results were inconclusive, excision biopsy was done for definitive diagnosis. Fluid specimens (pleural, cerebrospinal fluid, gastric aspirate, synovial) were subjected to microscopy, culture, Xpert MTB/RIF and biochemical analysis. Diagnosis of skin TB was more complex and consequently different tests were performed to ascertain the diagnosis including fluorescent microscopy, tissue culture and skin biopsy with histological analysis. The total duration of treatment for category-I (new patients) was 6 months whereas for category-II (relapse, lost to follow-up, and failure with previous TB treatment) the duration was 8 months as stated in national guidelines. Patients diagnosed with EPTB and PTB concomitantly were grouped under PTB based on NTP guidelines [10]. PTB refers to any bacteriologically confirmed or clinically diagnosed case of TB involving the lung parenchyma or the tracheobronchial tree. Miliary TB is classified as PTB because there are lesions in the lungs. EPTB refers to any bacteriologically confirmed or clinically diagnosed case of TB involving organs other than the lungs, e.g. pleura, lymph nodes, abdomen, genitourinary tract, skin, joints and bones, meninges [11]. According to WHO guidelines [9], patients who were stated “cured” and/or “completed treatment” were termed as “treatment success”, and all those patients who were lost to follow-up, died, or treatment failure were reported under a category of “unsuccessful treatment”. A *cured* patient was a bacteriologically confirmed PTB patient who became smear negative in the last month of treatment and on at least one previous occasion. *Treatment completed* included TB patients who completed treatment with no record to show smear negative in the last month of treatment and on at least one previous occasion.

## Results

### Proportion of childhood TB cases

From 1st June to 1st November 2016, childhood TB added up to 508 out of 2634 (19.3%) of all TB cases in five selected districts namely Hyderabad, Jamshoro and Matiari. All 508 patients met the inclusion criteria and were enrolled in the study. Overall, the highest numbers of children with TB (274; 54%) were registered in ICDK. In Hyderabad, the maximum numbers of patients were registered at LUMHS-CHH (145; 28.5%) compared to SGH-QH (15; 3%) and SBGH-LH, (22; 4.3%) whereas 52; 10.2% cases were reported in SBS-THM.

### Socio-demographic and baseline clinical characteristics of children with TB

The number and proportion of patients with TB during the study period are presented in Table 1. Of these, 268 (52.8%) were females and 240 (47.2%) were males with median age of 4 with interquartile range of 2–8 years. Based on the diagnostic results, 396 (78%) patients had pulmonary TB (PTB) and 112 (22%) had EPTB. Of EPTB cases, most cases (38.4%) had peripheral lymphadenitis. All sputum smear cases were repeated on Xpert MTB/RIF assay for further confirmation of *M. tuberculosis* in PTB- and resistance TB in PTB+. There were three cases which had negative results in sputum microscopy but were found positive on Xpert MTB/RIF test. Regarding therapeutic categorization, 485/508 (95.5%) children were enrolled as new cases while 23/508 (4.5%) were reported as retreatment cases. Seventy-two (14.2%) had no BCG scar. HIV test was done in all children and none were HIV-positive.

**Table 1:** Socio-demographic and clinical characteristics of children with TB in Hyderabad, Matiari and Jamshoro districts of Pakistan (*n* = 508)

Variables	*n* (%)
Gender	
Male	240 (47.2)
Female	268 (52.8)
Age (years)	
≤ 2	174 (34.2)
3–5	120 (23.6)
6–10	139 (27.4)
11–14	75 (14.8)
Residence	
Rural	206 (40.6)
Urban	302 (59.4)
Type of TB	
PTB+	29 (5.7)
PTB-	52 (10.2)
PTBNS	315 (62.0)
EPTB	112 (22.0)
EPTB site	
Peripheral lymph	
nodes	43 (38.4)
Abdominal	28 (25.0)
Pleural	14 (12.5)
Bones/Joints	1 (0.9)
Meningitis	22 (19.6)
Skin	4 (3.6)
Weight (percentiles)	
Underweight	457 (90.0)
Normal*	51 (10.0)
Registration category	
New	485 (95.5)
Retreated	23 (4.5)
BCG scar	
Present	436 (85.8)
Absent	72 (14.2)
Baseline CXR	
Normal	131 (25.8)
Abnormal	318 (62.6)
Not done	59 (11.6)
TST	
Positive (≥ 10 mm)	268 (52.8)
Negative (< 10 mm)	240 (47.2)

*< 5 percentiles, PTB- = smear negative pulmonary TB,

*PTB+* smear positive pulmonary TB, *PTBNS* pulmonary TB

with no sputum examination, *EPTB* extra-pulmonary TB,

*TST* Tuberculin Skin Test, and *BCG* Bacillus Calmette-Guerin

♣ include relapse, Lost to follow-up and failure patients with previous TB treatment

*CXR* Chest X-ray

### Baseline symptoms of TB in children

Cough (349/508, 68.7%) and weight loss (325/508, 64%) were the most frequently observed symptoms among the patients. Beside this, 277/508 (54.5%) patients had fever. Seizure (17/508, 3.3%), vomiting (18/508, 3.7%) and abdominal pain (21/508, 4.1%) were reported in patients with meningitis and abdominal TB. Caregivers of 188/508 (37%) of the patients complained of decreased physical activity.

### Laboratory values at baseline visit

The laboratory characteristics of the patients are summarized in Table 2. Twenty three percent and 36.8% of patients had increased lymphocyte and Erythrocyte Sedimentation Rate (ESR) value, respectively. The monocyte to lymphocyte ratio (MLR) was 0.23 ± 0.11 and Neutrophil to Lymphocyte Ratio (NLR) was 6.71 ± 0.16. During evaluation, a significant positive correlation was observed in NLR and ESR.

**Table 2:** Laboratory values of patients at baseline visit (*n* = 487)

Variables	Mean ± SD	*n* (%)
Hemoglobin		
Normal (13–16 g/dL)	8.9 ± 1.9	21 (4.3)
Below normal		466 (95.7)
RBCs		
Normal (3.8–5.9 millions/mm^3^)	4.15 ± 0.7	286 (58.7)
Below normal		178 (36.6)
Above normal		23 (4.7)
Lymhocytes		
Normal (20–45%)	32 ± 17.7	253 (52)
Below normal		121 (24.8)
Above normal		113 (23.2)
Monocytes		
Normal (02–10%)	7.4 ± 2.1	470 (96.6)
Below normal		8 (1.6)
Above normal		9 (1.8)
Neutrophils		
Normal (40–75%)	49.7 ± 19.4	270 (55.4)
Below normal		43 (8.8)
Above normal		174 (35.7)
ESR		
Normal (20 mm/hr)	37.32 ± 43	264 (52)
Above normal		187 (36.8)
Not available		57 (11.2)

### Frequency of EPTB among study participants

Of 112 cases of EPTB, the most common site of EPTB was the lymph nodes frequently seen among children aged 6 to 14 years old (Table 3). Those aged ≤2 years had higher proportion of meningitis. Females were presented with significantly higher cases of lymph node TB (40.7%) compared to other types of EPTB while frequency of abdominal and skin TB was higher in male patients. Residence, BCG scar and TST were significantly associated with types of EPTB.

**Table 3:** Types of EPTB among the study participants in the studied hospitals (*n* = 112)

Variables	Lymph node *n* = 43 (%)	Abdominal *n* = 28 (%)	Pleural *n* = 14 (%)	Bones/ Joints *n* = 1 (%)	Meningitis *n* = 22 (%)	Skin *n* = 4 (%)	Total cases *n* = 112	*P* value*
Gender								
Male	19 (35.8)	17 (32.1)	5 (9.4)	00	9 (17)	3 (5.7)	53 (47.3)	0.366
Female	24 (40.7)	11 (18.6)	9 (15.3)	1 (1.7)	13 (22)	1 (1.7)	59 (52.7)	
Age (years)								
≤ 2	6 (30)	3 (15)	2 (10)	00	9 (45)	00	20 (17.9)	0.105
3–5	7 (36.8)	5 (26.3)	1 (5.3)	00	5 (26.3)	1 (5.3)	19 (17)	
6–10	18 (38.3)	17 (36.2)	6 (12.8)	00	4 (8.5)	2 (4.3)	47 (42)	
11–14	12 (46.2)	3 (11.5)	5 (19.2)	1 (3.8)	4 (15.4)	1 (3.8)	26 (23.1)	
Weight (percentiles)								
Normal	11 (52.4)	4 (19)	2 (9.5)	00	3 (14.3)	1 (4.8)	21 (18.8)	0.765
Underweight**	32 (35.2)	24 (26.4)	12 (13.2)	1 (1.1)	19 (20.9)	3 (3.3)	91 (81.2)	
Residence								
Rural	14 (29.8)	8 (17)	6 (12.8)	1 (2.1)	16 (34)	2 (4.3)	47 (42)	0.018
Urban	29 (44.6)	20 (30.8)	8 (12.3)	00	6 (9.2)	2 (3.1)	65 (58)	
BCG scar								
Present	35 (36.5)	24 (25)	14 (14.5)	1 (1)	18 (18.8)	4 (4.2)	96 (85.7)	< 0.001
Absent	8 (50)	4 (25)	00	00	4 (25)	00	16 (14.3)	
Registration Category at baseline								
New	41 (38.3)	25 (23.4)	14 (13.1)	1 (0.9)	22 (20.6)	4 (3.7)	107 (95.5)	0.428
Retreated	2 (40)	3 (60)	00	00	00	00	5 (4.5)	
TST								
Positive (≥10 mm)	23 (53.5)	13 (30.2)	3 (7)	00	4 (9.3)	00	43 (38.4)	0.019
Negative (<10 mm)	20 (29)	15 (21.7)	11 (15.9)	1 (1.4)	18 (26.1)	4 (5.8)	69 (61.6)	

*Statistically significant values ≤ 0.05, **< 5 percentiles

### Association of socio-demographic and clinical characteristics with treatment outcomes

Summary of treatment outcomes is given in Table 4. Of the 508 children in the study, 483 (95.1%) achieved successful treatment outcomes with 90.7% of complete adherence to the treatment; 15 (3%) were cured and 468 (92.1%) completed treatment. Of those with unfavourable outcomes, 6 (1.2%) died, 4 (0.8%) failed treatment, 9 (1.8%) were lost to follow-up and 6 (1.2%) were transferred out to other units as MDR-TB. Of the retreatment patients 18 of 21(86%) completed 8 months of treatment, while 2 died. Of the new cases 465/481 (96.7%) completed their 6 months treatment regimen while 4 died.. Of the 6 patients who died, 1 died during the 1st month of treatment, 4 in 2nd month and 1 in 3rd month. As per decision of panel of physician and paediatricians, TB was the immediate cause of death in all 6 patients.

**Table 4:** Treatment outcomes of patients in the studied hospitals (*n* = 508)

Characteristics	Cured *n* = 15 (3%)	Completed *n* = 468 (92.1%)	Failed *n* = 4 (0.8%)	*Lost to follow-up* *n* = 9 (1.8%)	Died *n* = 6 (1.2%)	Transferred out *n* = 6 (1.2%)	Total cases 508	*P* value*
Gender								
Male	7 (2.9)	223 (92.9)	2 (0.8)	2 (0.8)	3 (1.3)	3 (1.3)	240 (47.2)	0.800
Female	8 (3)	245 (91.4)	2 (0.7)	7 (2.6)	3 (1.1)	3 (1.1)	268 (52.8)	
Age (years)								
≤ 2	00	167 (96)	00	3 (1.7)	4 (2.3)	00	174 (34.2)	< 0.001
3–5	00	118 (98.3)	1 (0.8)	1 (0.8)	00	00	120 (23.6)	
6–10	6 (4.3)	126 (90.6)	2 (1.4)	2 (1.4)	2 (1.4)	1 (0.7)	139 (27.4)	
11–14	9 (12)	57 (76)	1 (1.3)	3 (4)	00	5 (6.7)	75 (14.8)	
Residence								
Rural	7 (3.4)	185 (89.8)	2 (1)	7 (3.4)	4 (1.9)	1 (0.5)	206 (40.6)	0.114
Urban	8 (2.6)	283 (93.7)	2 (0.7)	2 (0.7)	2 (0.7)	5 (1.7)	302 (59.4)	
Weight (percentiles)								
Normal	3 (5.9)	43 (84.3)	00	2 (3.9)	1 (2)	2 (3.9)	51 (10)	0.165
Underweight**	12 (2.6)	425 (93)	4 (0.9)	7 (1.5)	5 (1.1)	4 (0.9)	457 (90)	
Type of TB								
PTB+	15	5 (17.2)	00	2 (6.9)	1 (3.4)	6 (20.7)	29 (5.7)	< 0.001
PTB-	(51.7)	48 (92.3)	3 (5.8)	1 (1.9)	00	00	52 (10.3)	
PTBNS	00	308 (98.1)	1 (0.3)	5 (1.6)	1 (0.3)	00	315 (62)	
EPTB	00	107 (95.5)	00	1 (0.9)	4 (3.6)	00	112 (22)	
Registration Category at baseline								
New	13 (2.7)	452 (93.2)	3 (0.6)	9 (1.9)	4 (0.8)	4 (0.8)	485 (95.5)	< 0.001
Retreated	2 (8.7)	16 (69.6)	1 (4.3)	00	2 (8.7)	2 (8.7)	23 (4.5)	
ADRs								
Yes	1 (1.5)	52 (77.6)	3 (4.5)	6 (9)	2 (3)	3 (4.5)	67 (13.2)	< 0.001
No	14 (3.2)	416 (94.3)	1 (0.2)	3 (0.7)	4 (0.9)	3 (0.7)	441 (86.8)	
BCG scar								
Present	14 (3.2)	398 (91.3)	3 (0.7)	9 (2.1)	6 (1.4)	6 (1.4)	436 (85.8)	< 0.001
Absent	1 (1.4)	70 (97.2)	1 (1.4)	00	00	00	72 (14.2)	
Baseline X-ray								
Normal	2 (1.5)	123 (93.9)	2 (1.5)	3 (2.3)	00	1 (0.8)	131 (25.8)	0.352
Abnormal	13 (4.1)	287 (90.3)	2 (0.6)	5 (1.6)	6 (1.9)	5 (1.6)	318 (62.6)	
Not done	00	58 (98.3)	00	1 (1.7)	00	00	59 (11.6)	
HHC with TB								
Yes	4 (3)	117 (86.7)	3 (2.2)	4 (3)	3 (2.2)	4 (3)	135 (26.6)	0.031
No	11 (2.9)	351 (94.1)	1 (0.3)	5 (1.3)	3 (0.8)	2 (0.5)	373 (73.4)	
TST								
Positive (≥ 10 mm)	12 (4.5)	247 (92.2)	1 (0.4)	5 (1.9)	2 (0.7)	1 (0.4)	268 (52.8)	0.082
Negative (< 10 mm)	3 (1.3)	221 (92.1)	3 (1.3)	4 (1.7)	4 (1.7)	5 (2.1)	240 (47.2)	
Monthly income of family (PKRs)								
< 5000 6000–10,000	1 (8.3) 6 (3.9)	9 (75) 142 (92.8)	00 1 (0.7)	2 (16.7) 1 (0.7)	00 1 (0.7)	00 2 (1.3)	12 (2.4) 153 (30.1)	0.123
11,000–20,000	6 (2.4)	233 (92.1)	2 (0.8)	4 (1.6)	4 (1.6)	4 (1.6)	253 (49.8)	
> 20,000	2 (2.2)	84 (93.3)	1 (1.1)	2 (2.2)	1 (1.1)	00	90 (17.7)	
Education of caregiver								
No formal education	10 (4.2)	211 (89)	3 (1.3)	7 (3)	2 (0.8)	4 (1.7)	237 (46.6)	< 0.001
Primary	2 (1.6)	119 (96.7)	00	2 (1.6)	00	00	123 (24.2)	
Secondary	2 (2.6)	74 (94.9)	1 (1.3)	00	1 (1.3)	00	78 (15.4)	
College	1 (2.8)	33 (91.7)	00	00	1 (2.8)	1 (2.8)	36 (7.1)	
Graduation	00	31 (91.2)	00	00	2 (5.9)	1 (2.9)	34 (6.7)	

*PTB*- smear negative pulmonary TB, *PTB+*: smear positive pulmonary TB, *PTBNS*: pulmonary TB with unknown sputum, *EPTB*: extra-pulmonary TB, PKRs: Pakistan rupees, * statistically significant value ≤0.05, **< 5 percentiles, HHC: household contacts

### Risk factors for unsuccessful treatment outcomes

Variables which showed statistically significant association with unsuccessful treatment outcomes in univariate analysis (Table 5) were age group 11 to 14 years (COR = 3.554, 95%CI *=* 1.50–8.37), PTB+ (COR = 13.022, 95%CI *=* 5.13–33.04), retreatment cases (COR = 6.458, 95%CI *=* 2.17–19.15), had ADRs (COR = 10.326, 95%CI *=* 4.45–23.91), patients with known TB contacts (COR = 3.808, 95%CI *=* 1.68–8.61) and caregivers with no formal education (COR = 4.903, 95%CI *=* 1.81–13.27).

**Table 5:** Logistic regression analysis of risk factors for unsuccessful treatment outcomes at the study site (*n* = 508)

Variables	Treatment outcomes		Univariate analysis	*P* value*	Multivariate analysis	*P* value*
	Successful *n* (%)	Unsuccessful *n* (%)	COR (95% CI)		AOR (95% CI)	
Gender						
Male	230 (95.8)	10 (4.2)	1	0.485	–	–
Female	253 (94.4)	15 (5.6)	1.364 (0.60–3.09)			
Age (years)						
≤ 2	167 (96)	7 (4)	1	0.501	–	–
3–5	118 (98.3)	2 (1.7)	0.269 (0.06–1.15)	0.078	–	–
6–10	132 (95)	7 (5)	1.034 (0.42–2.53)	0.942	–	–
11–14	66 (88)	9 (12)	3.554 (1.50–8.37)	0.004	2.908 (0.78–10.77)	0.110
Residence						
Rural	192 (93.2)	14 (6.8)	1.929 (0.85–4.33)	0.112	–	–
Urban	291 (96.4)	11 (3.6)	1			
Type of TB						
PTB+	20 (69)	9 (31)	13.022 (5.13–33.04)	< 0.001	5.910 (1.64–21.29)	0.007
PTB-	48 (92.3)	4 (7.7)	1.726 (0.56–5.23)	0.335	–	–
PTBNS	308 (97.8)	7 (2.2)	0.221 (0.09–0.53)	0.001	0.771 (0.24–2.43)	0.657
EPTB	107 (95.5)	5 (4.5)	1	0.800	–	–
Weight(percentiles)						
Underweight	46 (90.2)	5 (9.8)	2.375 (0.85–6.62)	0.098	–	–
Normal	437 (95.6)	20 (4.4)	1			
Registration category at baseline						
New	465 (95.9)	20 (4.1)	1	0.001	0.368 (0.08–1.56)	0.176
Retreated	18 (78.3)	5 (21.7)	6.458 (2.17–19.15)		2.720 (0.63–11.59)	
ADRs						
Yes	53(79)	14 (21)	10.326 (4.45–23.91)	< 0.001	11.601 (4.06–33.12)	< 0.001
No	430 (97.5)	11 (2.5)	1		0.086 (0.03–0.24)	
BCG scar						
Present	412 (94.5)	24 (5.5)	1	0.168	–	–
Absent	71 (98.6)	1 (1.4)	0.242 (0.03–1.81)			
Baseline X-ray						
Normal	125 (95.4)	6 (4.6)	1	0.834		
Abnormal	300 (94.3)	18 (5.7)	1.569 (0.64–3.82)	0.323	–	–
Not done	58 (98.3)	1 (1.7)	0.305 (0.04–2.30)	0.249		
TST						
Positive (≥ 10 mm)	259 (96.6)	9 (3.4)	1	0.091	–	–
Negative (< 10 mm)	224 (93.3)	16 (6.7)	2.056 (0.89–4.74)			
HHC with TB						
Yes	121 (89.6)	14 (10.4)	3.808 (1.68–8.61)	0.001	3.128 (1.21–8.06)	0.018
No	362 (97.1)	11 (2.9)	1		0.320 (0.12–0.82)	
Monthly income of family (PKRs)						
< 5000	10 (83.3)	2 (16.7)	4.113 (0.85–19.86)	0.078		
6000–10,000	148 (96.7)	5 (3.3)	0.566 (0.20–1.53)	0.264		
11,000–20,000	239 (94.5)	14 (5.5)	1.299 (0.57–2.92)	0.526	–	–
> 20,000	86 (95.6)	4 (4.4)	1	0.818		
Education of caregiver						
No formal education	217 (91.6)	20 (8.4)	4.903 (1.81–13.27)	0.002	0.623 (0.11–3.30)	0.579
Primary	121 (98.4)	2 (1.6)	0.260 (0.06–1.12)	0.071	–	–
Secondary	76 (98.7)	1 (1.3)	0.223 (0.03–1.67)	0.145	–	–
College	35 (94.6)	2 (5.4)	1	0.888	–	–
Graduation	34 (100)	00	–	–	–	–

PTB-: smear negative pulmonary TB, PTB+: smear positive pulmonary TB, *PTBNS*: pulmonary TB with unknown sputum, *EPTB*: extra-pulmonary TB, *COR*: crude odd ratio, *AOR*: adjusted odd ratio, PKRs: Pakistani rupees, *ADRs*: Adverse drug reactions, * Statistically significant value ≤ 0.05, *HHC*: household contacts

In multivariate analysis (Table 5), pulmonary smear positive TB (PTB+) (AOR = 5.910, 95%CI *=* 1.64–21.29), those with adverse drug reactions (AOR = 11.601, 95%CI *=* 4.06–33.12) and those who had known TB contact (AOR = 3.128, 95%CI *=* 1.21–8.06) showed statistically significant association with unsuccessful treatment outcomes.

### Follow-up results

Body weight was recorded for all patients at the follow-up visits. The dose of of the drugs were adjusted according to the weight gain. The Majority of children (95.1%) were documented to have weight gain to more or equal to the 5th percentile. At the end of intensive phase sputum smear examination was repeated for PTB+ and for them sputum conversion rate was recorded as 80% (23/29). All patients had their CXR examination repeated at the end of the continuation phase. Those who had normal CXR were recorded with successful treatment outcomes together with improved symptoms.

## Discussion

This study is one of few that report data on the patient’s profiles and treatment outcomes of TB patients aged ≤14 years enlisted and treated under DOTS in Pakistan. Nineteen percent of total number of TB cases were recorded for childhood TB at the study site. More than half of the cases (57.9%) were aged ≤5 years and 90% of children were noted underweight. The study findings however identify certain priority areas that need to be addressed by the National and State health authorities. Majority of participants in the present study were females (52.8%) but the difference was not significant.

The highest cases of TB in current study were observed among the children aged ≤2 years (34.2%) and this is in agreement with a previous study conducted in South Africa [12]. The higher rates in young children could be due to increased risk of developing disease after infection compared to older children, a high risk of being infected at a young age in a population with a high TB incidence is common in developing countries such as Pakistan. The extent of EPTB in current study was 22% and the most frequent site of EPTB in the present study was the lymph nodes, similar to the findings among children in previous studies [13, 14]. Meningitis was mainly observed in children < 5 years, this agrees with the study conducted among children in South Africa where 82% of meningitis cases were seen in children aged < 5 years [15].

A remarkable proportion of participants (90%) in our study were underweight. Malnutrition is a predefined risk factor for TB in children [16]. Children with TB are by and large observed to be malnourished, and it is common among all ages in Pakistan. Unfortunately, one third of children < 5 years in Pakistan are underweight, 44% stunted and 15% are wasted [17]. Around 32.5% of family in the present study had a monthly income of ≤10,000 in Pakistan rupees (PKRs) (approximately 75 US dollars), whereas, 50% reported to have income of ≤20,000 PKRs, demonstrating the very poor and poor status of families, respectively. In addition to poverty, education of parents may have impact on child’s health and care, as 46.6% of caregivers (particularly mothers) of the study cohorts had no formal education. Studies have recognized statistically significant relationship between child malnutrition and mother’s education and economic status of family [18, 19]. At the baseline visit, high proportion (95.7%) of patients had a hemoglobin level below normal. Haematological anomalies had been used as a diagnostic marker in TB [20]. Presence of anemia in TB patients is a notable fact, documented in number of studies [21, 22]. High proportion of anemia in the current cohort could possibly be related to their socio-economical background. Additionally, patients in the current study were reported with lymphocytopenia and lymphocytosis indicating active TB. Lymphocytopenia specifies TB infection whereas lymphocytosis point towards inflammatory process in TB patients [23, 24]. Previously, the neutrophil count in TB have been positively correlated with increased bacillary count [25]. Nevertheless, in this study neutrophilia was positively associated with CXR abnormalities including bilateral infiltrations and cavitary lesions representing active TB likewise justified by Lyadova [26] where increased neutrophil count represents progression of infection to active TB. Further detailed studies are required to correlate the present hematologic findings in TB children with large sample.

Overall successful treatment outcome (cured and treatment complete) was recorded as 95.1%. Hence, the study site reached the targeted treatment success rate of 85% for TB under DOTS set by WHO [27]. Comparable treatment rates for children treated under DOTS have been reported from Ethiopia (85.5%) [28], South Africa (89.5%) [29] and Iran (91.7%) [30]. Treatment success rate in the current cohort of patients was comparatively better than that reported from Malawi (45, 77.3%) [31, 32], Democratic Republic of Congo (59%) [33], Botswana (67%) [34], Ethiopia (77, 78.9%) [35, 36], Nigeria (79.2%) [37], Tanzania (79.9%) [38].

In the present study, 9 (1.8%) patients were lost to follow-up. Of these, 33.3% were lost to follow-up during first two months and the remaining 66.7% during the continuous phase of the treatment. Lost to follow-up rate in the study participants was well below the range reported for children in previous studies in different places 5.1–21.1% [29–31, 33, 36–38]. The low lost to follow-up rate observed in the current cohort could be accredited to the programmatic efforts at the study site to lessen the barriers to care including regular counseling on monthly visits, persistent supply of drugs and tracing patients on phone in case of delay in scheduled monthly visits. Significantly higher lost to follow-up cases were observed in children 11 to 14 years, PTBNS and retreatment cases.

In our study, 6 (1.2%) patients died during treatment. The mortality rate in our study is consistent with results of earlier studies (0.7, 1.4 and 1.8%) [28, 29, 33] but lower than the rate reported in literature [31, 32, 35, 37–39]. This could be because initiation of treatment was frequently seen within first 2 days of diagnosis. Timely commencement of treatment has been established as the main predictive factor that determines disease fatality and consequences [40]. Significantly increased deaths were reported in children aged < 2 years similar to the study conducted in Africa [29] and those from rural areas. Because of immature immune system, children especially under 2 years are at a higher risk of death from infectious diseases including TB. Additionally, TB such as TB meningitis is associated with high mortality and is more frequent among young children [39]. Comparing the mortality in patients with types of TB, the proportion was higher in EPTB with maximum death cases in meningitis (50%). The proportions of patients with treatment failure in our study (0.8%) are comparable with the previous studies conducted elsewhere [28–30, 33]. Failure of PTB- and PTBNS patients was alarming in this study and may indicate misdiagnosis or incorrect entry of patient.

On multivariate analysis, children with PTB+, ADRs and those had HHC with TB were significant risk factors for unsuccessful treatment outcomes.. Contact with an individual suffering from TB is an important risk factor for TB in children. This factor was significantly associated with unsuccessful treatment outcomes in the present study which is consistent with a previous study [33]. In Iran, household contact was found as the main risk factor for poor treatment outcome in children [30]. Total 135 (26.6%) children were recorded with household contacts of TB. Of these, 56 (41.5%) had completed ATT in last 2 years and 79 (58.5%) were still under treatment. Seven of source cases had MDR-TB, 27 had PTB- and 44 were registered as PTB+ with majority of sputum grading + 2. Presence of MDR-TB and PTB+ could have increased the severity of disease in children and increased the risk of poor treatment outcomes in them.

### Limitations

The findings of this study consolidated information of patients with complete information of their treatment outcome at five different hospitals. TB patients transferred to other health facilities were not followed until the end of treatment. In addition, important patient information which could influence TB treatment outcome, including co-morbidity with other chronic illnesses, distance from the treatment center, and adherence level of the caregivers for their child’s treatment were not collected and thus not included in the analysis. Sputum microscopy is done in very limited number of children. Furthermore, gastric lavage to acquire sputum among young children for smear microscopy are not frequently used at the study site. Hence, these constraints need to be considered while interpreting the findings.

## Conclusion

The high proportion of childhood TB cases (19.3%) indicates a high rate of TB transmission in the study area. Additionally, noticeable number of children with TB was underweight. Keeping in mind to end-up the malnutrition, we recommend that concerned health authorities along with policy stakeholders should take initiatives to control poor nutrition, accelerate the efforts to add fundamental supplements to diet with TB treatment. The risk factors for unsuccessful treatment outcomes in the present study are generally distinguishable before diagnosis or ahead in the course of ATT. TB epidemic cannot be controlled by treating just the active cases with ATT.

## Abbreviations

ADRs: Adverse drug reactions
AFB: Acid-Fast Bacilli
AORs: Adjusted odds ratios
ATT: Anti-tuberculosis treatment
BCG: Bacillus Calmette-Guerin
CDC: Centres for Disease Control and Prevention
CIs: Confidence intervals
COR: Crude odds ratios
CXR: Chest X-ray
DOTS: Directly observed therapy strategy
EPTB: Extra-pulmonary TB
ESR: Erythrocyte sedimentation rate
FNAC: Fine Needle Aspiration Cytology
*M. tuberculosis*: *Mycobacterium tuberculosis*
MLR: Monocyte to lymphocyte ratio
NLR: Neutrophil to lymphocyte ratio
NTP: National Tuberculosis Control Program
PKRs: Pakistan rupees
PPA: Pakistan Pediatric Association
PTB: pulmonary TB
PTB: smear negative PTB
PTB +: smear positive PTB
PTBNS: Pulmonary TB with unknown sputum
RBC: Red blood cells
TB: Tuberculosis
TST: Tuberculin skin test
WHO: World Health Organization
Xpert MTB/RIF: Gene Xpert MTB/RIF
